# P53 and aging: A fresh look at an old paradigm

**DOI:** 10.18632/aging.100179

**Published:** 2010-07-16

**Authors:** Masha V. Poyurovsky, Carol Prives

**Affiliations:** Department of Biological Sciences, Columbia University, New York, NY 10017

Apoptosis and cellular senescence, two key tumor suppression mechanisms, are thought to be antagonistically pleiotropic. Antagonistic pleiotropy holds that functions that are advantageous for a young and reproductively fit organism (eg. cancer protection and proper development) can be deleterious when that same organism becomes old (eg. loss of stem cell proliferation and tissue degeneration leading to diseases associated with age) [1]. This theory predicts that in an older animal (or human), the activity of tumor suppressors would be associated with enhanced aging phenotypes. However, confirmation of a direct connection between apoptosis, senescence and aging remains elusive [2]. In fact, at least in the case of p53 there is mounting data challenging the antagonistic pleiotropy model.

Multiple lines of evidence from animal models suggest that a functional p53 pathway favors prolonged survival. Aging mice show a decrease in p53 activity correlated with increased tumor incidence as well as an overall reduction in longevity [3]. On the other hand, mice with an extra gene dosage of Arf and p53 show significant tumor protection, decreased oxidative damage and delayed aging [4]. Animals expressing the p53^S18A^ mutation present with accelerated aging, and cells from these mice undergo early senescence [5].  As phosphorylation of p53 at Ser18 (Ser15 in humans) is associated with activation, these results highlight a requirement for intact p53 signaling in longevity [6]. In the nematode*Caenorhabditis elegans*, mutations that lead to longevity preferentially antagonize tumor growth, likely due to an increase in DAF-16/p53-dependentapoptosis [7].

The mTOR pathway is intimately connected with organismal aging. In fact inhibition of mTOR either by treatment with rapamycin or by the inhibition of upstream signaling molecules, extends lifespan in yeast, worms and flies suggesting that  this pathway may  be one of the main mechanisms that decrease lifespan [8]. p53 is able to regulate activity of mTOR following DNA damage or oncogenic stress by activation of PTEN, AMP kinase and TSC-2, each of which signals to diminish the activity of mTOR (Figure 1) [9,10]. p53 may also function downstream of mTOR by activating antioxidant genes and thus protecting cells against increased ROS levels in cells, one of the consequences of heightened mTOR activity [11-13].

As the ability of p53 to increase longevity becomes more evident, we should consider the role of its negative regulator, Mdm2, if not in the process of aging directly, at least in its effects on the activity of mTOR. Growth factor and oncogene signaling activate PI-3 kinase and its downstream effector AKT, a protein kinase that activates mTOR via inhibition of TSC-1 protein.  AKT also phosphorylates Mdm2 leading to enhanced Mdm2 ubiquitin ligase activity and more rapid degradation of p53 (Figure 1) [8,14]. Additionally, AKT stimulates FOXO phosphorylation, which results in FOXO nuclear exportation and ubiquitin dependent proteasomal degradation [15]. FOXO proteins have conserved abilities to increase longevity in worms and flies [16]. Indeed, Mdm2 was reported to function as an E3 ubiquitin ligase to promote FOXO degradation, following activation of AKT, thus forging an additional link between Mdm2 and changes in longevity [17].

Additionally, mTOR is able to positively regulate Mdm2 through an increase in translation of Mdm2 mRNA. Consistently, an increase in p53 dependent apoptosis in the liver of mouse embryos treated with Rapamycin *in utero* is attributed mTOR's ability to control translation of Mdm2 [18]. However, because the interplay between apoptosis and aging is likely to be highly context specific, it is important to note that mice expressing only ~30% of the wild type Mdm2 levels do not have an aging phenotype, while exhibiting clear enhancement in tumor protection [19].  It would be of interest to look at the effects of nutrient deprivation on the mice carrying an Mdm2 hypomorphic allele.

**Figure 1.**
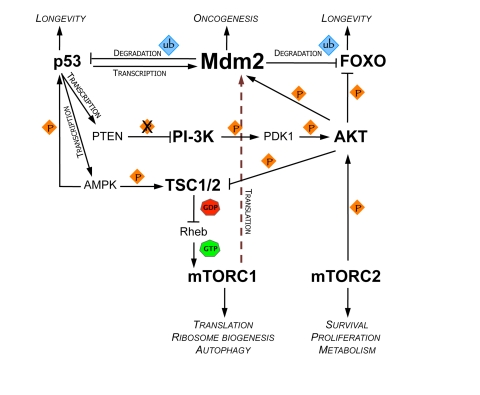
Signaling circuitry connecting Mdm2 with the regulation of longevity and metabolism. Both mTORC1 and 2 (mammalian target of rapamycin complex 1/2) are able to positively regulate the activity of Mdm2, either through enhancement of translation of Mdm2 mRNA or via activation of AKT. Conversely, Mdm2 can activate mTORC1 by targeting p53 for degradation.  p53 negatively regulates mTORC1 by activating TSC1/2 (tuberous sclerosis 1/2) complex, which acts as a GTPase activating protein (GAP) for Rheb (Ras homologue enriched in brain). p53 can also repress the activity of PI-3K (phosphatidylinositol 3-kinase) by induction of PTEN (phosphatase and tensin homologue), leading to further downregulation of mTORC1. Arrows represent up-regulation. Orange diamonds represent kinase activity. Blue diamonds represent E3 ubiquitin ligase activity of Mdm2. AMPK, AMP-activated protein kinase; FOXO, Forkhead box; PDK1, 3-phosphoinositide-dependent protein kinase 1.

In conclusion, as our notion of p53 function in aging and senescence changes, it is very tempting to imagine that just a slight inhibition of Mdm2 function in cells could both prolong full tumor surveillance mechanisms of p53, and in some circumstances increase longevity. Numerous molecular inhibitors of Mdm2 are in various stages of development with the goal of reactivating p53 activity in cancer [20]. The idea that controlled pharmacological modulation of Mdm2 function might also have positive consequences in extension of human lifespan could be an unexpected benefit and an additional incentive for design of new compounds targeting Mdm2.

## References

[1] Campisi J (2005). Aging, tumor suppression and cancer: high wire-act. Mech Ageing Dev.

[2] Johnson FB, Sinclair DA, Guarente L (1999). Molecular biology of aging. Cell.

[3] Feng Z, Hu W, Teresky AK, Hernando E, Cordon-Cardo C, Levine AJ (2007). Declining p53 function in the aging process: a possible mechanism for the increased tumor incidence in older populations. Proc Natl Acad Sci U S A.

[4] Matheu A, Maraver A, Klatt P, Flores I, Garcia-Cao I, Borras C, Flores JM, Vina J, Blasco MA, Serrano M (2007). Delayed ageing through damage protection by the Arf/p53 pathway. Nature.

[5] Armata HL, Garlick DS, Sluss HK (2007). The ataxia telangiectasia-mutated target site Ser18 is required for p53-mediated tumor suppression. Cancer Res.

[6] Bode AM, Dong Z (2004). Post-translational modification of p53 in tumorigenesis. Nat Rev Cancer.

[7] Pinkston JM, Garigan D, Hansen M, Kenyon C (2006). Mutations that increase the life span of C. elegans inhibit tumor growth. Science.

[8] Hands SL, Proud CG, Wyttenbach A (2009). mTOR's role in ageing: protein synthesis or autophagy. Aging (Albany NY).

[9] Levine AJ, Feng Z, Mak TW, You H, Jin S (2006). Coordination and communication between the p53 and IGF-1-AKT-TOR signal transduction pathways. Genes Dev.

[10] Demidenko ZN, Korotchkina LG, Gudkov AV, Blagosklonny MV (2010). Paradoxical suppression of cellular senescence by p53. Proc Natl Acad Sci U S A.

[11] Hu W, Zhang C, Wu R, Sun Y, Levine A, Feng Z (2010). Glutaminase 2, a novel p53 target gene regulating energy metabolism and antioxidant function. Proc Natl Acad Sci U S A.

[12] Suzuki S, Tanaka T, Poyurovsky MV, Nagano H, Mayama T, Ohkubo S, Lokshin M, Hosokawa H, Nakayama T, Suzuki Y, Sugano S, Sato E, Nagao T, Yokote K, Tatsuno I, Prives C (2010). Phosphate-activated glutaminase (GLS2), a p53-inducible regulator of glutamine metabolism and reactive oxygen species. Proc Natl Acad Sci U S A.

[13] Bensaad K, Cheung EC, Vousden KH (2009). Modulation of intracellular ROS levels by TIGAR controls autophagy. EMBO J.

[14] Ogawara Y, Kishishita S, Obata T, Isazawa Y, Suzuki T, Tanaka K, Masuyama N, Gotoh Y (2002). Akt enhances Mdm2-mediated ubiquitination and degradation of p53. J Biol Chem.

[15] Aoki M, Jiang H, Vogt PK (2004). Proteasomal degradation of the FoxO1 transcriptional regulator in cells transformed by the P3k and Akt oncoproteins. Proc Natl Acad Sci U S A.

[16] Kenyon C (2005). The plasticity of aging: insights from long-lived mutants. Cell.

[17] Fu W, Ma Q, Chen L, Li P, Zhang M, Ramamoorthy S, Nawaz Z, Shimojima T, Wang H, Yang Y, Shen Z, Zhang Y, Zhang X, Nicosia SV, Pledger JW, Chen J, Bai W (2009). MDM2 acts downstream of p53 as an E3 ligase to promote FOXO ubiquitination and degradation. J Biol Chem.

[18] Moumen A, Patane S, Porras A, Dono R, Maina F (2007). Met acts on Mdm2 via mTOR to signal cell survival during development. Development.

[19] Mendrysa SM, O'Leary KA, McElwee MK, Michalowski J, Eisenman RN, Powell DA, Perry ME (2006). Tumor suppression and normal aging in mice with constitutively high p53 activity. Genes Dev.

[20] Vassilev LT (2004). Small-molecule antagonists of p53-MDM2 binding: research tools and potential therapeutics. Cell Cycle.

